# Supplementary material for: The phage-encoded PIT4 protein affects multiple two-component systems of Pseudomonas aeruginosa
Source: Microbiol Spectr. 2023 Nov 14;11(6):e02372-23. doi: 10.1128/spectrum.02372-23 (PMC10714779; doi:10.1128/spectrum.02372-23)
Supplement: Supplemental material — Fig. S1 to S7; Tables S1 to S4. [file spectrum.02372-23-s0001.docx]

##
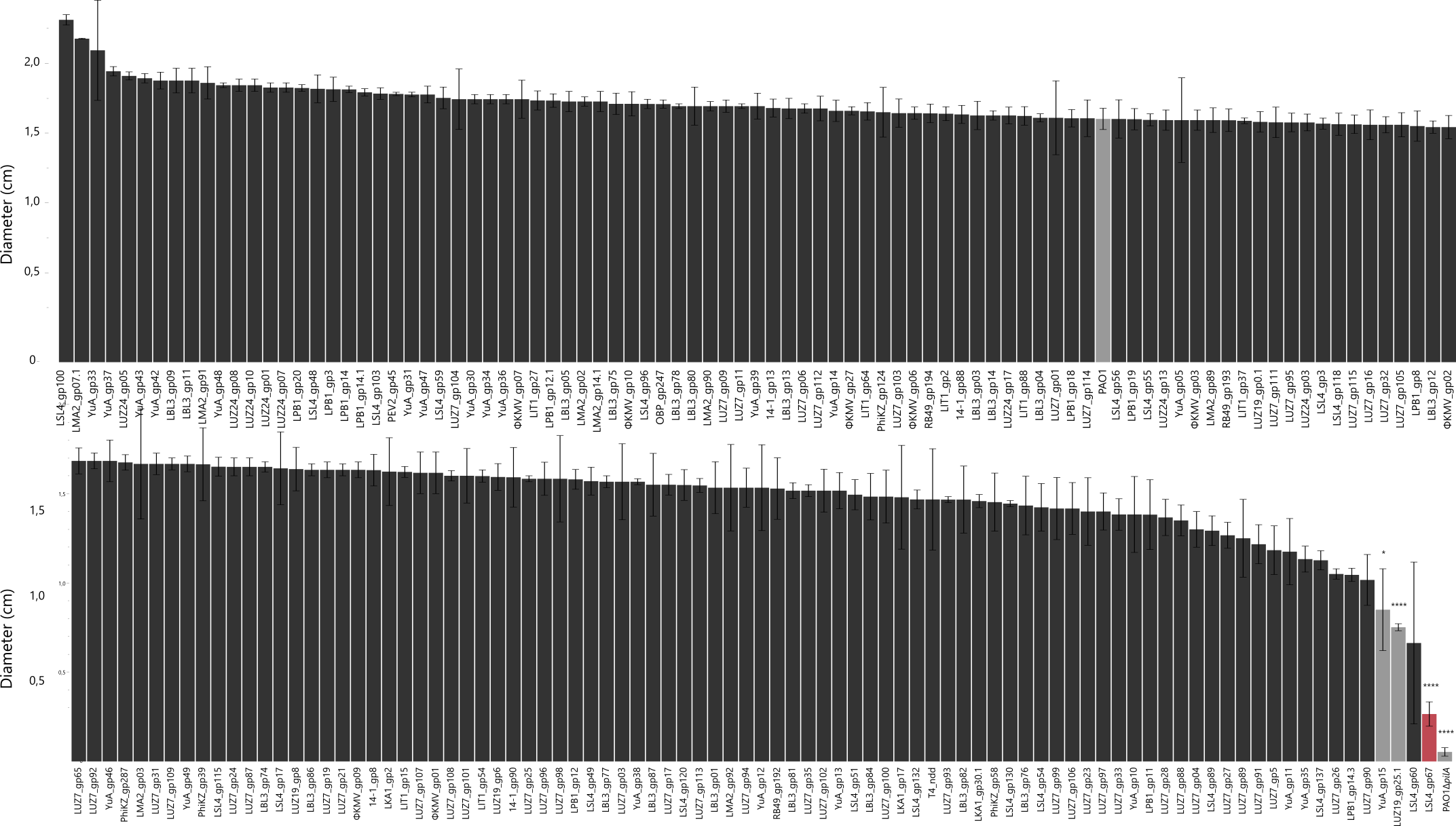
Supplemental Data

**Figure S1: High-throughput screening of the effect of phage-derived proteins on the twitching motility of PAO1.** The effect of the production of a phage-derived protein in the bacterial PAO1 cell was assessed by measuring the resulting twitching diameter after 24 hours. Out of this screen, three phage proteins (LSL4gp67, LUZ19gp25.1 and YuAgp15, indicated in grey) showed to significantly reduce the bacterial twitching motility (Student’s t-test, **** = p < 0.0001 and * = p < 0.05)


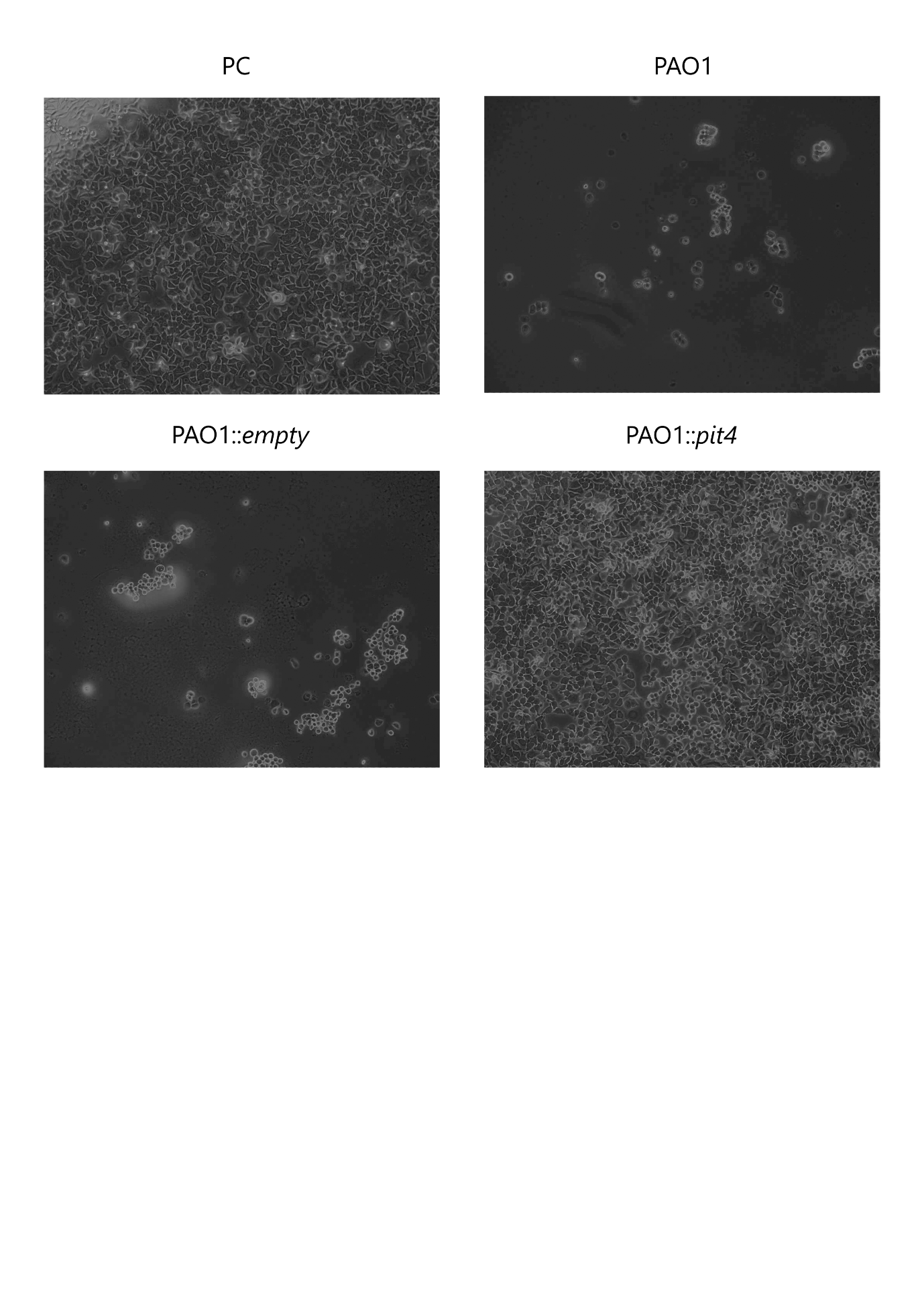


**Figure S2: Microscopic data of the HeLa cells treated with different bacterial strains.** The cells of the positive control (PC), HeLa cells in growth medium, showed to be viable when observed under the microscope. In contrast, the majority of the cells treated with PAO1 and PAO1 harboring an empty construct, were death or detached from the bottom of the plate. HeLa cells exposed to PAO1 cells producing the PIT4 protein appeared to have the same viability as the positive control.


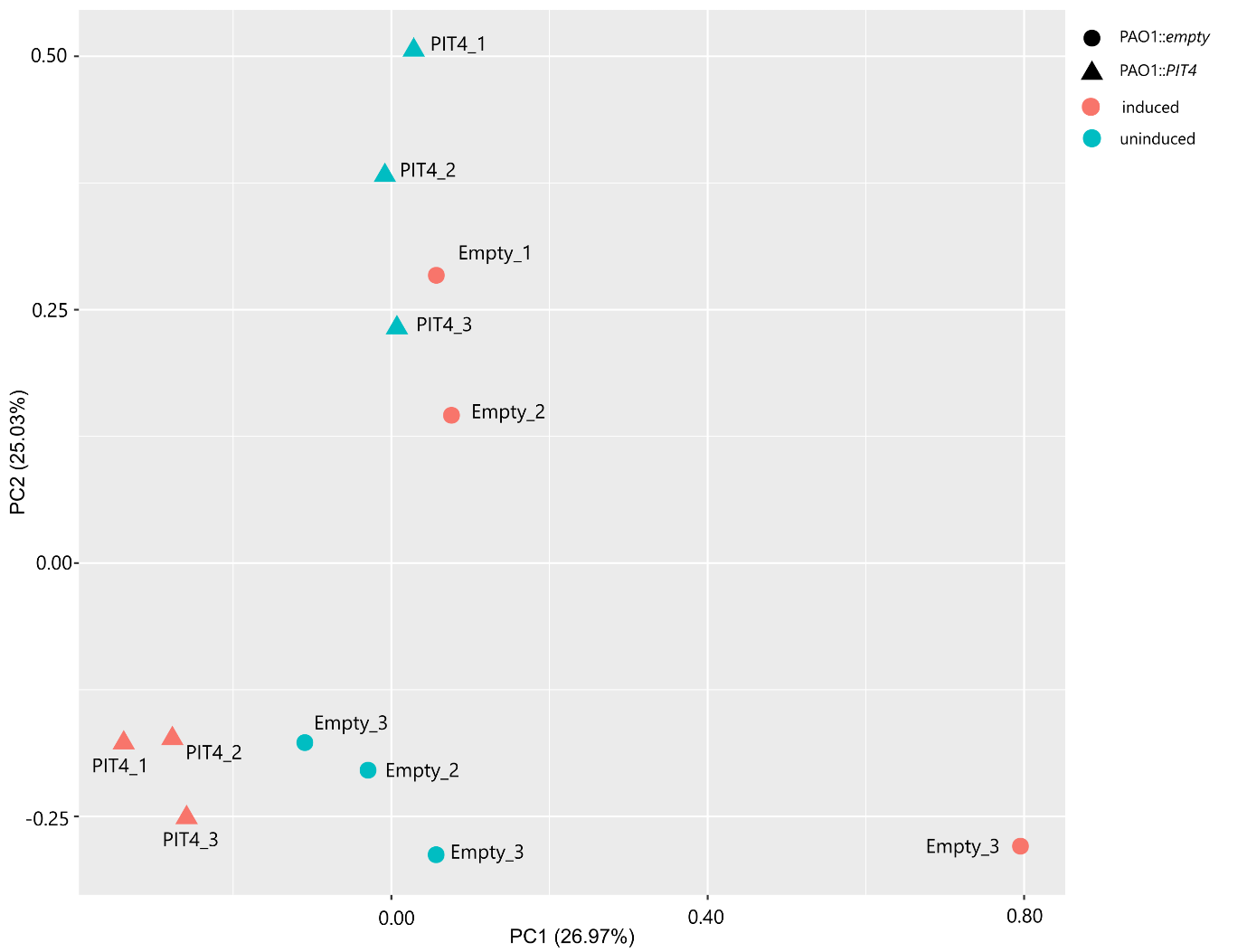


**Figure S3: Principal component analysis of the differential gene expression.** A clustering of the different conditions is observed, which indicates a different expression profile between the different strains and/or conditions. The number after the underscore resembles number of the replicate.

**Table S1: Differential expressed genes under PIT4 producing conditions.** Log2FoldChange > |1.2| and p_adj_ < 0.001. With hp = hypothetical protein.

| Locus tag | Gene name | Log2Fold change | p_adj_ |
| --- | --- | --- | --- |
| PA1183 | *dctA* | -4.748992925 | 5.71E-188 |
| PA4581.1 | *tRNA - Arg* | -2.79571024 | 1.62E-25 |
| PA3662 | *hp* | -2.439641043 | 5.08E-54 |
| PA1077 | *flgB* | -2.418976311 | 2.13E-94 |
| PA5167 | *dctP* | -2.347524282 | 2.92E-111 |
| PA4586 | *hp* | -2.326343567 | 2.16E-12 |
| PA1081 | *flgF* | -2.323249909 | 9.26E-77 |
| PA5169 | *dctM* | -2.269910094 | 4.25E-100 |
| PA1079 | *flgD* | -2.161233255 | 1.96E-80 |
| PA5267 | *hcpB* | -2.128686234 | 1.49E-75 |
| PA0172 | *siaA* | -2.113970961 | 2.90E-23 |
| PA5168 | *dctQ* | -2.058522678 | 4.36E-53 |
| PA4844 | *ctpL* | -2.048639375 | 1.17E-11 |
| PA3526 | *motY* | -2.047144528 | 1.43E-77 |
| PA4582 | *hp* | -2.039578781 | 5.53E-63 |
| PA4843 | *gcbA* | -2.03097084 | 3.82E-124 |
| PA1512 | *hcpA* | -2.01029023 | 1.29E-45 |
| PA1078 | *flgC* | -2.007804044 | 7.93E-41 |
| PA2463 | *hp* | -1.992375308 | 1.34E-39 |
| PA1913 | *hp* | -1.986094782 | 1.85E-42 |
| PA4683 | *hp* | -1.981634798 | 2.06E-17 |
| PA5266 | *vgrG6* | -1.978012491 | 1.74E-33 |
| PA1911 | *femR* | -1.976982478 | 0.000235359 |
| PA0268 | *hcpC* | -1.885965821 | 1.03E-30 |
| PA5072 | *mcpK* | -1.885654542 | 8.36E-55 |
| PA4585 | *rtcA* | -1.878520628 | 5.00E-24 |
| PA1679 | *hp* | -1.853443517 | 9.39E-57 |
| PA1967 | *hp* | -1.822920769 | 1.54E-39 |
| PA1080 | *flgE* | -1.816423712 | 1.17E-82 |
| PA0320 | *carO* | -1.785543511 | 1.01E-19 |
| PA0671 | *hp* | -1.760964482 | 2.38E-09 |
| PA3386 | *hp* | -1.748147983 | 2.64E-22 |
| PA0714 | *hp* | -1.723666511 | 4.14E-18 |
| PA2381 | *hp* | -1.723054909 | 1.41E-82 |
| PA4583 | *hp* | -1.720999812 | 6.59E-51 |
| PA1441 | *fliK* | -1.719227771 | 1.30E-84 |
| PA1132 | *hp* | -1.695379489 | 1.78E-28 |
| PA5383 | *hp* | -1.692729115 | 3.19E-07 |
| PA2426 | *pvdS* | -1.691758023 | 0.00030599 |
| PA1545 | *hp* | -1.688767895 | 2.38E-39 |
| PA3928 | *hp* | -1.626745371 | 2.45E-39 |
| PA1123 | *hp* | -1.620495137 | 1.91E-75 |
| PA3367 | *hp* | -1.610616417 | 2.12E-07 |
| PA5357 | *hp* | -1.608831696 | 1.51E-35 |
| PA3385 | *amrZ* | -1.604759246 | 9.72E-62 |
| PA4520 | *hp* | -1.561215465 | 5.06E-67 |
| PA2788 | *hp* | -1.559745623 | 1.55E-76 |
| PA3722 | *hp* | -1.558518629 | 7.09E-35 |
| PA4525 | *pilA* | -1.525575787 | 2.25E-88 |
| PA3930 | *cioA* | -1.521874545 | 1.22E-63 |
| PA1912 | *femL* | -1.512314348 | 1.17E-09 |
| PA3929 | *cioB* | -1.510638876 | 4.31E-71 |
| PA2288 | *hp* | -1.49705 | 6.22E-16 |
| PA5530 | *hp* | -1.48468 | 2.95E-08 |
| PA3720 | *hp* | -1.4826 | 4.94E-17 |
| PA4865 | *urea* | -1.47707 | 7.18E-11 |
| PA0169 | *siaD* | -1.47333 | 8.51E-12 |
| PA1680 | *hp* | -1.47173 | 1.09E-06 |
| PA2311 | *hp* | -1.4668 | 0.00023552 |
| PA4306 | *flp* | -1.45653 | 3.48E-33 |
| PA2686 | *pfeR* | -1.45268 | 8.18E-15 |
| PA0713 | *hp* | -1.45079 | 1.98E-47 |
| PA0171 | *siaB* | -1.44992 | 2.02E-12 |
| PA4599 | *mexC* | -1.44608 | 2.37E-07 |
| PA3307 | *hp* | -1.44541 | 1.17E-12 |
| PA4864 | *ureD* | -1.42998 | 7.81E-05 |
| PA2867 | *hp* | -1.4237 | 9.56E-66 |
| PA2462 | *hp* | -1.42343 | 2.34E-67 |
| PA0800 | *hp* | -1.42154 | 5.59E-06 |
| PA4219 | *ampO* | -1.41781 | 7.32E-06 |
| PA4929 | *hp* | -1.41262 | 5.41E-32 |
| PA4307 | *pctC* | -1.39038 | 1.46E-55 |
| PA0170 | *siaC* | -1.38495 | 5.08E-06 |
| PA4310 | *pctB* | -1.36494 | 5.37E-64 |
| PA2652 | *hp* | -1.35572 | 5.43E-48 |
| PA4524.1 | *tRNA-Thr* | -1.34964 | 7.02E-32 |
| PA3530 | *bfd* | -1.32106 | 2.01E-07 |
| PA4324 | *hp* | -1.30614 | 1.92E-42 |
| PA4515 | *hp* | -1.30056 | 3.03E-23 |
| PA0952 | *hp* | -1.29401 | 2.52E-13 |
| PA4221 | *fptA* | -1.28137 | 1.61E-32 |
| PA4601 | *morA* | -1.27541 | 1.48E-36 |
| PA4226 | *pchE* | -1.27013 | 1.48E-31 |
| PA0670 | *hp* | -1.26452 | 5.13E-07 |
| PA4863 | *hp* | -1.2594 | 7.06E-18 |
| PA3069 | *hp* | -1.25585 | 4.10E-23 |
| PA4218 | *ampP* | -1.25348 | 9.99E-09 |
| PA0472 | *fivI* | -1.24344 | 3.39E-15 |
| PA4228 | *pchD* | -1.2302 | 1.01E-20 |
| PA0612 | *ptrB* | -1.22808 | 1.17E-12 |
| PA4633 | *hp* | -1.21175 | 1.56E-53 |
| PA2561 | *ctpH* | -1.20297 | 9.95E-14 |
| PA2648 | *nuoM* | 1.203067 | 4.53E-44 |
| PA2406 | *fpvK* | 1.229945 | 0.000139735 |
| PA2407 | *fpvC* | 1.252719 | 2.96E-11 |
| PA2410 | *fpvF* | 1.253916 | 2.23E-17 |
| PA5170 | *arcD* | 1.265748 | 3.84E-44 |
| PA2138 | *ligD* | 1.340905 | 3.07E-08 |
| PA0102 | *hp* | 1.386964 | 2.14E-47 |
| PA2398 | *fpvA* | 1.398194 | 2.07E-38 |
| PA2403 | *fpvG* | 1.487694 | 6.99E-29 |
| PA3441 | *hp* | 1.490148 | 0.000124407 |
| PA2408 | *fpvD* | 1.508222576 | 6.60E-07 |
| PA2828 | *hp* | 1.551293319 | 4.64E-39 |
| PA0090 | *clpV1* | 1.563830054 | 2.53E-63 |
| PA2405 | *fpvJ* | 1.565890064 | 5.08E-15 |
| PA0113 | *hp* | 1.762407259 | 2.94E-23 |
| PA0091 | *vgrG1* | 1.928347086 | 6.86E-94 |
| PA1844 | *tse1* | 1.929553408 | 2.53E-11 |
| PA2404 | *fpvH* | 1.937230284 | 1.35E-28 |
| PA2409 | *fpvE* | 1.946408691 | 1.09E-14 |
| PA0103 | *hp* | 2.016911972 | 2.74E-51 |
| PA1190 | *hp* | 2.01787453 | 1.33E-49 |
| PA0112 | *hp* | 2.12412003 | 6.29E-17 |
| PA1845 | *tsi1* | 2.132842384 | 9.11E-29 |
| PA0108 | *coIII* | 2.318873692 | 1.49E-87 |
| PA0110 | *hp* | 2.39725323 | 2.97E-50 |
| PA0105 | *coxB* | 2.574769666 | 8.30E-212 |
| PA0111 | *hp* | 2.610436891 | 1.72E-26 |
| PA0106 | *coxA* | 2.656530827 | 1.55E-204 |
| PA0107 | *hp* | 2.976158225 | 2.52E-81 |


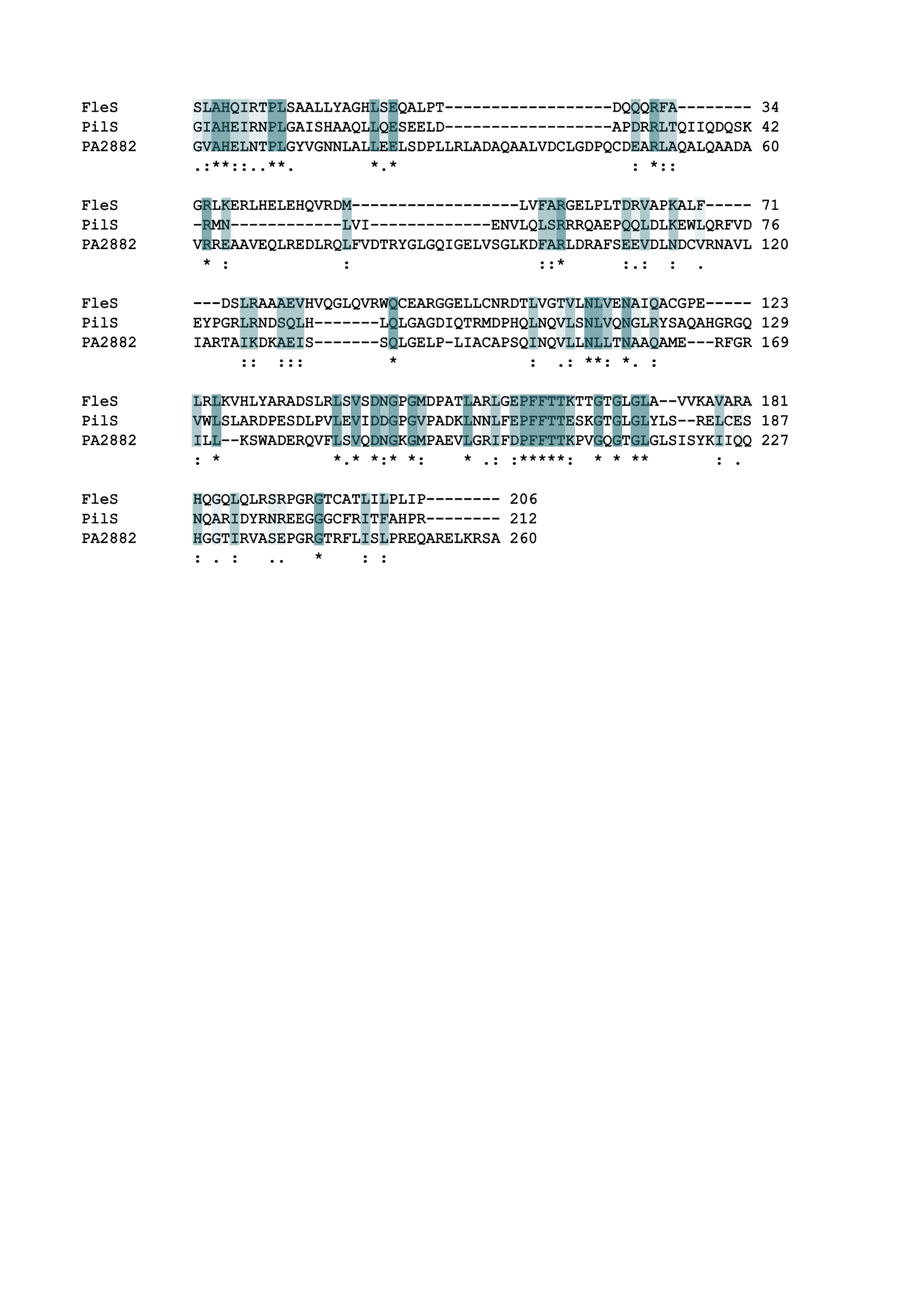


**Figure S4: Multiple sequence alignment (ClustalOmega) of the histidine kinase (HK) domains of FleS, PilS and PA2882.** Darker shaded background colors indicate a higher level of conservation with * = fully conserved residu for the three sequences, : = residues with strongly similar properties (> 0.5 in the Gonnet PAM 250 matrix) and = residues with weakly similar properties (< 0.5 in the Gonnet PAM 250 matrix). A 28 % identity was found between the HK domains of FleS and PilS, 31 % and 29 % identity between the HK domains of FleS and PA2882 and PilS and PA2882, respectively. With the fourth residue of each protein the conserved histidine (H), which is responsible for the kinase activity.


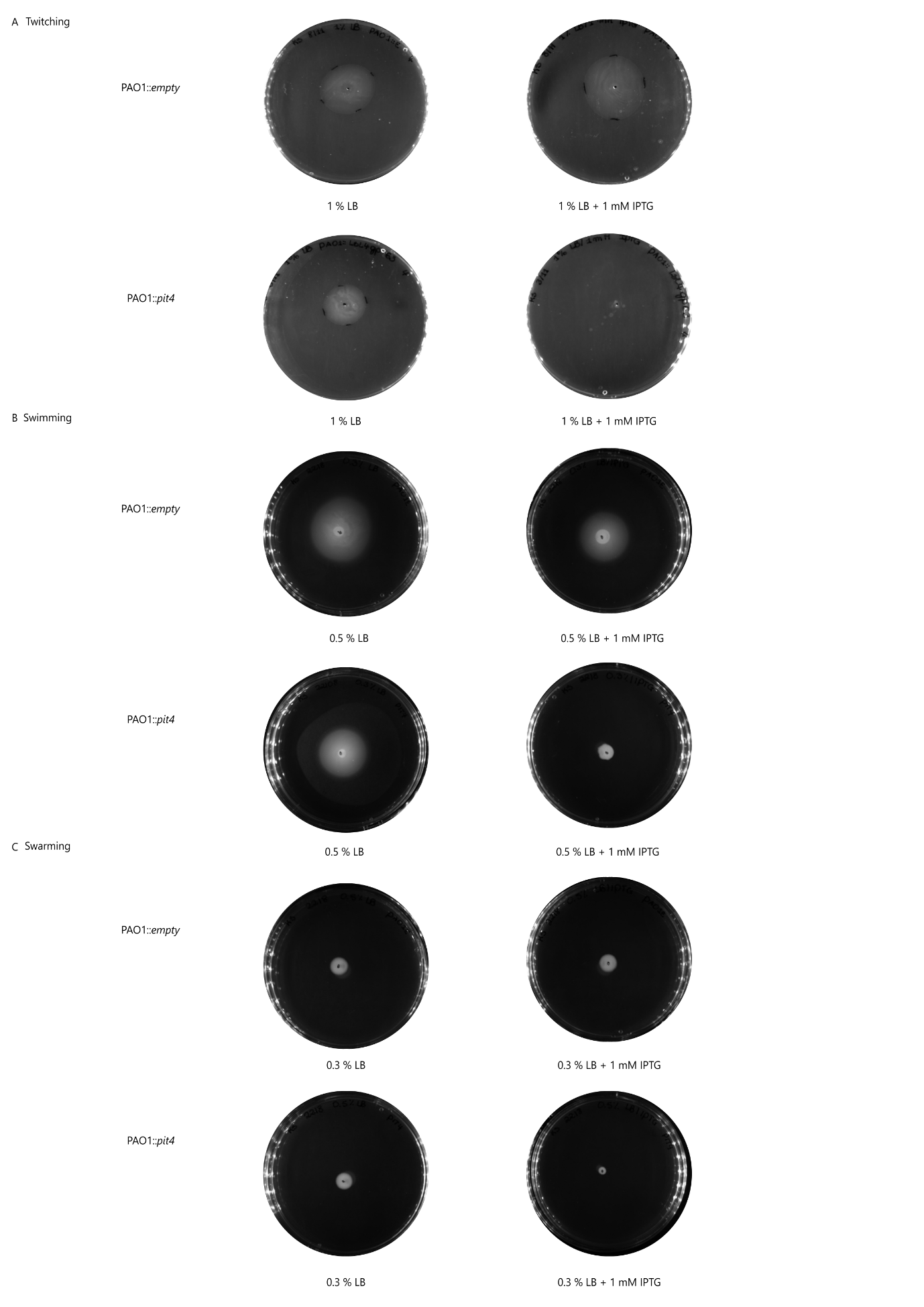


**Figure S5:Motility diameters of PAO1 strains on plate.** (A) When PAO1 cultures are stabbed in a 1 % LB plate, bacterial twitching motility can be observed. Remarkably, upon expression of PIT4, twitching motility is completely abolished. (B) Swimming motilities were determined by dropping the PAO1 cultures on a 0.5 % LB plate. A reduced swimming motility is noticed upon PIT4 production. (C) By dropping the PAO1 cultures on a 0.3 % plate, swarming motility was determined. No swarming is seen in case of pit4 expression. . Pictures were taken on different days

*
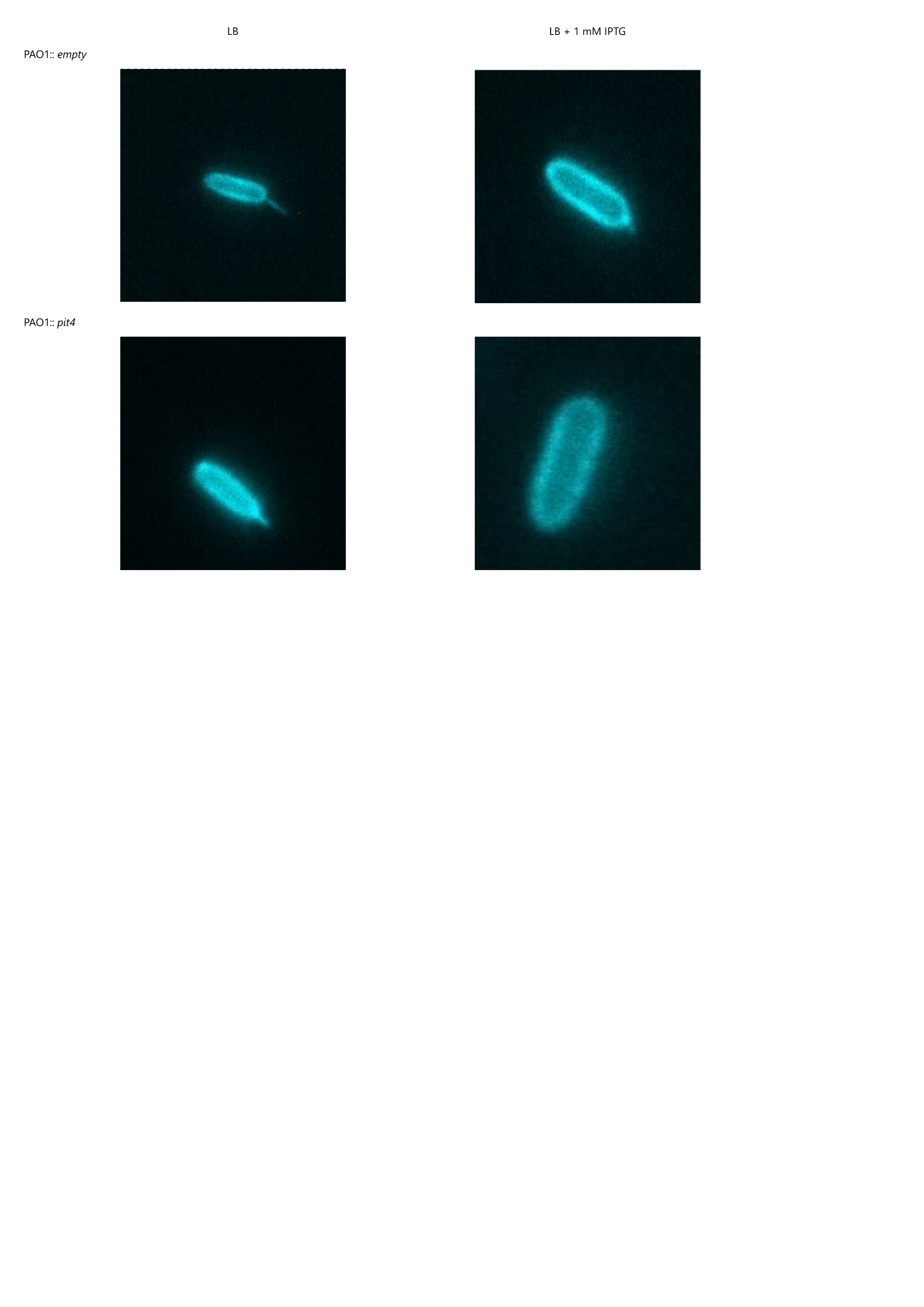
*

**Figure S6: Fluorescent microscopy images.** The T4P present on the bacterial surface were visualized via fluorescent microscopy. Two different strains, PAO1::empty and PAO1::pit4 were imaged in two different conditions (LB and LB + 1 mM IPTG). Fifty cells were assessed for each condition and the number of displayed T4P was determined. Interestingly, no T4P are being displayed

**Table S2: The average number of motility moieties counted on the surface of the bacterial strains.** For each condition, 40 different cells were observed with a transmission electron microscope and the number of pili-like structures were counted. No significant differences could be seen in the average number of displayed pili structures per cell between the control (PAO1::empty) and the strains producing PIT4 (One-way analysis of variance, p = 0.5212). Conversely, a significant difference in the average number of flagella per cell was seen between PAO1::*empty* and PAO::*pit4* upon induction (Student’s, t-test, p > 0.0001).

| Strain | IPTG (mM) | pili structures/ cell | Flagella/ cell |
| --- | --- | --- | --- |
| PAO1::*empty* | 1 | 0.55 (± 0.14) | 0.75 (± 0.07) |
| PAO1::*pit4* | 0 | 0.65 (± 0.14) | 0.67 (± 0.07) |
|  | 1 | 0.43 (± 0.14) | 0.32 (± 0.07) |


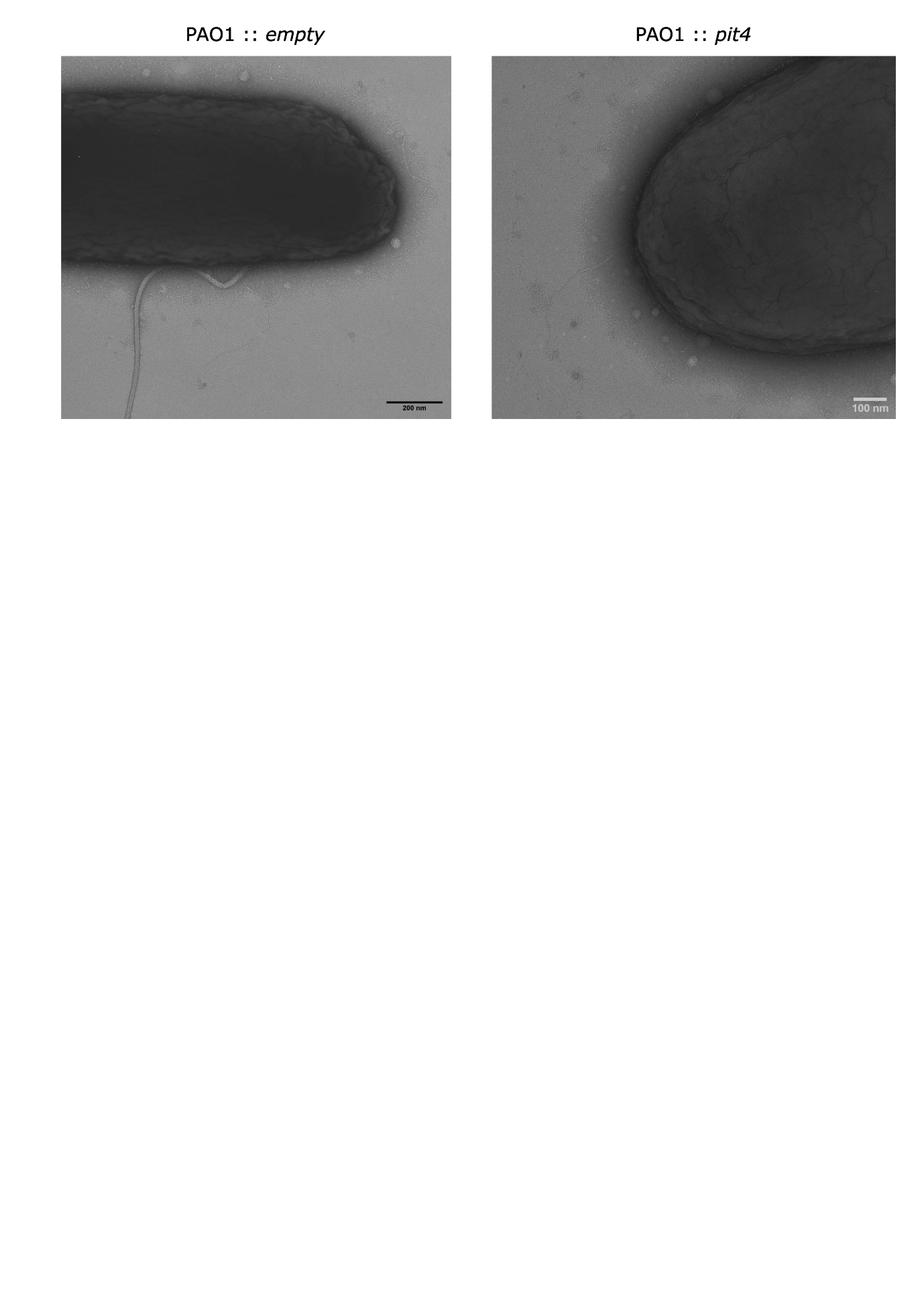


**Figure S7: Transmission electron microscopy (TEM) reveals the presence of the T4P.** When PIT4 is produced inside the PAO1 cell, no significant difference in the number of displayed T4P on the bacterial surface compared to the control (PAO1:: empty) is noticed.

**Table S3: List of primers used in this work**

| Name | Sequence |
| --- | --- |
|  | |
| **Introduction of *pit4* in pBGDes** | |
| pBG_BsaI_F | GACGGTCTCTTAACGAATTCGAGCTCGGTAC |
| pBG13_BsaIPEM_R | GTCGGTCTCCCATTTAGAAAACCTCCTTAGCATGATTAAGATG |
| ST_PIT4_F | ATAGCTCTTCCAGAGGTCTCCAATGAGCGAAATCAACGTTTATCCCAGC |
| ST_PIT4_R | GGCGCTCTTCACTTGGTCTCCGTTAAATTTGCTCATGGTACTTCTCCTG |
|  |  |
| **PIT4 production** |  |
| PIT4_Strep_F | TTATTTTTCGAACTGCGGGTGGCTCCAAGCGCTAATTTGCTCATGGTACTTC |
| PIT4_Strep_R | ATGAGCGAAATCAACGTTTATCCCAGCATTGCTGAGAATATTTACCCTGGACTGACC |
| PIT4_pET28_F | GCTCAGTGGTGGTGGTGGTGGTGCTCGAGTTATTTTTCGAACTGCGGGTG |
| PIT4_pET28_R | GTTTAACTTTAAGAAGGAGATATACCATGGATGAGCGAAATCAACGTTTATCCCAGCATT |
|  |  |
| **Quality control RNA samples** | |
| GlmS_up | GTGCGACTGCTGGAGCTGAA |
| GlmS_down | GCTCTCGCCGATCCTCTACA |
|  |  |
| **B2H plasmids** | |
| pUT18_gp63_F | GCTTGCATGCCTGCAGGTCGACTCTAGAAGGAGATACCCTTATGAGCGAAATCAACG |
| pUT18_gp63_R | GGCCTCGCTGGCGGCTGAATTCGGAATTTGCTCATGGTACTTCTCCTG |
| pUT18C_gp63_F | GGCGCAGTGGAACGCCACTGCAGGAGCGAAATCAACGTTTATC |
| pUT18C_gp63_R | GAGCTCGGTACCCGGGGATCCTCTAGATTTCAAATTTGCTCATGGTACTTCTCC |
| pKT25_FleS_F | GGGCTGCAGGGTCGACTCTAGAACAACCAGCCCTCAACGCC |
| pKT25_FleS_R | CGTTGTTGTAAAACGACGGCCGAATTCGCTTACTCCTGAATCGCAGAAAGAGGCG |
| pKNT25_FleS_F | CGGCTGCAGGGTCGACTCTAGAAGGAGATACCCTTATGCAACCAGCCCTCAACG |
| pKNT25_FleS_R | GATTGCTGCATGGTCATTGAATTCGGCTCCTGAATCGCAGAAAGAGGC |
| pKT25_PilS_F | GGGCTGCAGGGTCGACTCTAGATCGCGCTGAACGGCTACG |
| pKT25_PilS_R | CACGACGTTGTAAAACGACGGCCGAATTCCGTCAGCTGAGTTTGCGTGG |
| pKNT25_PilS_F | CGGCTGCAGGGTCGACTCTAGAAGGAGATACCCTTATGCGCGCTGAACGGCTAC |
| pKNT25_PilS_R | GGAATTCGAGCTGAGTTTGCGTGGGTGGGC |
| pKT25_2882__F | GGGCTGCAGGGTCGACTCTAGATCAACGCTTCGGCCGTGAC |
| pKT25_2882__R | TTGTAAAACGACGGCCGAATTCAATCACGCACTACGTTTCAGCTCG |
| pKT25_HK_F | GCTGCAGGGTCGACTCTAGAATCGCTGGCCCACCAGATC |
| pKT25_HK_R | TTCTAGAGTCGACCCTGCAGCCCGCCG |
|  |  |
| **Y2H plasmids** | |
| PIT4_Y2H_F | GATCAGAATTCATGAGCGAAATCAACGTTTATCCCAGC |
| PIT4_Y2H_R | GATGGATCCTCAAATTTGCTCATGGTACTTCTCCTG |
| pGBT9_MSC_F | GCCTCTAACATTGAGACAGCATAG |
| pGBT9_MSC_R | TGAGAAAGCAACCTGACCTACAG |
|  |  |
| **PAO1(*pilA*^A86C^)** |  |
| PilA_spacer_F | GAAACTACTGCGACCGAAACATATGTCGGCGTCGAGCCGG |
| PilA_spacer_F | GCGACCGGCTCGACGCCGACATATGTTTCGGTCGCAGTAG |
| PilA86_BsaI_UpF | GACGGTCTCCTGGCTTGGACGAGCTGTCGG |
| PilA86_BsaI_UpR | GTCGGTCTCCACGCCGACATATGTTTCGGTCGCAGTAGAGGCAGTAGTAC |
| PilA86_BsaI_DwnF | GACGGTCTCCGCGTCGAGCCGGATTGTAACAAGTTGGGTG |
| PilA86_BsaI_DwnR | GTCGGTCTCCCGTCGACTACATCTCCATCGGCAC |
| PilA86_SEVA_F | GACGGTCTCCGACGACTAGTCTTGGACTCCTG |
| PilA86_SEVA_R | GTCGGTCTCCGCCATTAATTAAAGGCATCAAATAAAACG |
| PilA86_chk_F | TGCTCGGCGGACAATTC |
| PilA86_chk_R | TCATGCTCGACGAACTCAG |
|  |  |

**Table S4: Sequence of constructed gene block used in this work**

| Name | Sequence |
| --- | --- |
|  |  |
| PA2882 | CGGCTGCAGGGTCGACTCTAGAAGGAGATACCCTTATGCAACGCTTCGGCCGTGACTTATGGGGCCGTCATCCCGTGCGTGACGTGATGAACCGCGCGCCCCTGAGTGTTTCTCTTGGAGCCTCACTTGAGGAAGCTGCTCAACAGGTTACCGGGCGTCTTCAATACCCTATTACGGAAGATTTCGCGCTGGTAGATGAAGAGGGTCGTTATCGCGGACTGGGCACTGTTCTGGATCTGCTGAAAGCAATGGAGGCCCGCATTGCTCAACGCAACCGCGTCTTGCGTAAAGCACTTGTCGATCTGAAAGAAAGCCAAGCGCAACTTGTGCAGTCGGAAAAAATGGCTAGCTTGGGTCAGATGGTGGCGGGAGTTGCCCATGAATTGAATACTCCACTGGGATACGTGGGGAACAATCTTGCGCTTTTAGAGGAACTGTCCGATCCACTTTTACGTTTAGCAGACGCGCAAGCAGCTTTGGTCGATTGCTTAGGAGACCCGCAGTGCGACGAGGCACGTTTAGCGCAGGCTTTACAGGCGGCCGACGCAGTACGCCGTGAAGCCGCTGTCGAACAGTTACGTGAAGATCTTCGCCAGTTGTTTGTTGACACTCGTTACGGGCTTGGACAAATTGGAGAACTTGTTAGTGGTTTAAAAGACTTCGCACGTCTTGACCGCGCGTTCTCGGAGGAAGTAGATCTTAACGACTGCGTGCGTAACGCGGTTCTTATTGCCCGTACGGCAATCAAGGACAAGGCTGAAATTTCATCTCAACTTGGAGAGCTTCCATTGATTGCCTGCGCCCCATCACAGATTAACCAGGTCCTGTTGAACTTACTGACCAATGCGGCACAGGCTATGGAACGTTTCGGCCGTATTTTATTGAAATCTTGGGCTGACGAACGTCAAGTTTTTTTAAGCGTACAGGACAATGGTAAAGGAATGCCGGCTGAAGTTTTGGGACGCATCTTTGATCCATTTTTTACGACTAAACCTGTTGGTCAAGGCACTGGGCTGGGGTTGAGCATCTCCTACAAGATTATCCAACAGCATGGGGGGACGATTCGCGTTGCTAGCGAGCCTGGCCGCGGGACTCGCTTTCTGATCAGCTTACCCCGTGAGCAAGCACGCGAGCTGAAACGTAGTGCGTTGAATTCAATGACCATGCAGCAATC |
